# Society and Post-Graduate Programmes

**Published:** 1952-01

**Authors:** 


### *Society and Post-Graduate Programmes*

It is not always possible to give up-to-date information about future meetings, etc., because programmes are not always completed in time to be included. A list of medical societies and secretaries has, therefore, been given at the end of this news. Some regular informal meetings held in Bristol at which visitors will be welcome are included. The editor will be glad to receive corrections and additions.

### BRISTOL MEDICO-CHIRURGICAL SOCIETY

Jan. 9th. Dr. C. D. Evans, " **Contact Dermatitis**".

Feb. 13th. Dame Louise McIlroy, "**The Prevention and Treatment of Post-Partum Haemorrhage**".

Mar. 12th. Dr. Beryl Corner, " **Life for the Premature Baby.**"

Apr. 9th. Mr. R. E. Horton, " **Appendicitis in Childhood** ".

May 14th. Dr. Faulkner Hudson, " **The Value of Work in the Treatment of Disability** ".

### B.M.A. BRISTOL DIVISION

Jan. 16th. Dr. MacDonald Critchley, " **The Body Image** ". B.M.A. Lecture.

Apr. 16th. Dr. Frazer Roberts, " **Genetics** ".

June 18th. Clinical meeting.

### B.M.A. GLOUCESTERSHIRE BRANCH

Feb. 14th, Cheltenham. Mrs. Blanche Percival, M.B.E., " **Marriage Guidance and Social Health** ".

### WEST OF ENGLAND CHILD HEALTH GROUP. (PROVISIONAL PROGRAMME)

Meetings at Bristol, Jan. 31st, Feb. 28th, Mar. 22nd, April 24th.

Jan. 31st, at 5 p.m. Dr. E. E. Mawson, " **Mass Radiography in Childhood** ".

Feb. 28th at 5 p.m. Dr. R. F. Barbour, " **Enuresis—physical and psychiatric aspects** ".

Mar. 22nd at 2.45 p.m. Clinical Meeting, Children's Hospital.

Apr. 24th at 5 p.m. Mr. Ramsay Garden, " **Defective Vision in Childhood—Medical and Social Aspects** ".

May. Meeting in Gloucestershire, date, time and place to be arranged.

Hon. Secretary, Dr. R. C. Wofinden, Central Health Clinic, Bristol.

### BRISTOL SOUTH GROUP OF GENERAL PRACTITIONERS

Meetings on the first Sunday of each month at 8 p.m. (October–June) in the board room, Bristol General Hospital.

Hon. Secretary, Dr. D. M. Cameron, Lindthorpe, 1 Dean Lane, Southville, Bristol 3.

### COSSHAM MEDICAL SOCIETY

Meetings on the first Thursday at 9 p.m. (October—March) with two summer meetings.

Hon. Secretary, Dr. I. Macdonald, 43 Regent Street, Kingswood, Bristol.

### DEVON AND EXETER MEDICO-CHIRURGICAL SOCIETY

Jan. 17th. Mr. R. Hinde, "**The Surgical Restoration of Hearing**".

Feb. 7th. Dr. G. M. Colson, "**The Scope of Clinical Electro-cardiography**".

Feb. 21st. Mr. K. S. Powell, "**What is new in Anaesthesia**".

Mar. 6th. Dr. M. C. Binnie, "**Geriatrics**".

Mar. 20th. Dr. Stewart Smith and Dr. Warren.

Apr. 3rd. Mr. H. K. Griffith, "**The Surgical Treatment of Bronchiectasis**".

Apr. 17th. Meeting at Hawkmoor Sanatorium.

### PLYMOUTH MEDICAL SOCIETY

Jan. 11th, at 8.30 p.m. Professor A. M. Boyd, "**Peripheral Vascular Disease**".

Jan. 25th, at 7.30 p.m. Meeting at R.N. Hospital.

Feb. 15th, at 8.30 p.m. Mr. N. R. Barrett, "**Oesophagitis**".

Mar. 14th, at 8.30 p.m. Clinical Evening.

Apr. 9th, at 2.30 p.m. Clinical Demonstration.

Hon. Secretary, Mr. W. B. Waterfall, 2 Crescent Villas, Athenaeum Street, Plymouth.

### THE CLINICAL SOCIETY OF BATH

Meetings on January 12th, February 9th, March 9th, April 13th, at Royal United Hospital at 8.15 p.m.

### TORQUAY AND DISTRICT MEDICAL SOCIETY

(*Meetings at Torbay Hospital unless stated otherwise.*)

Jan. 24th, at 8.30 p.m. Dr. J. Franklin, "**The Modern Approach to some Dermatological Problems**".

Feb. 14th, at 8.30 p.m. Professor G. R. Cameron, "**Cortisone and A.C.T.H.**".

Feb. 28th, at 8.30 p.m. Dr. R. F. Barbour, "**Difficult Behaviour in Children**".

Mar. 13th, (Newton Abbot Hospital at 4.30 p.m.) Clinical Meeting.

Mar. 27th, at 8.30 p.m. Dr. Keith Simpson, "**Crime Reconstruction**".

June 12th (R.N.C. Dartmouth at 3 p.m.) Summer Meeting.

Hon. Secretary, Dr. R. A. Lattey, Ambrook, Rousdown Road, Torquay.

### REGULAR MEETINGS AT BRISTOL AT WHICH VISITORS WILL BE WELCOME

(The name and telephone number of the organizer is given in brackets.)

*Southmead Hospital.* **Obstetrics.** Every Thursday at 5 p.m. Discussion of cases. (Professor G. G. Lennon, Bristol 68974.) **Gastroenterology.** Second Friday at 4.30 p.m. in X-ray Department. (Dr. J. M. Naish, c/o Medical Typists, Southmead: Bristol 68031.) **Radiology.** Every Wednesday in term, at 5 p.m. in X-ray Department. (Dr. J. V. Sparks or Dr. G. R. Airth: Bristol 68031.)

*Canynge Hall.* Every Tuesday in term, at 5.15 p.m. Clinical-pathological conference. (Professor T. F. Hewer, Bristol 38257.)

*Barrow Hospital.* Every Monday at 5 p.m. Discussion of cases. (Dr. R. E. Hemphill, 85 3162/3.)

### POST-GRADUATE LECTURES, ETC.

*Bath. Royal National Hospital.* May 9th–11th. Course in Rheumatic Diseases, (apply to Dr. G. D. Kersley).

*Bristol University.* It is intended to arrange courses for general practitioners in Paediatrics and in Chest Diseases on Sunday mornings in April–June. Details later from Director Medical Post-graduate Studies, The University, Bristol 8. (Bristol 24161, Ext. 7.)

*Royal Devon and Exeter Hospital.* A series of lectures for general practitioners is generally arranged on Tuesdays at 8.30 p.m. in the months October–December. Details from Dr. A. Daly, 13 Southernhay West, Exeter.

*West Cornwall Hospital, Penzance.* (Under the auspices of the West Penwith Medical Society). Sunday morning rounds are arranged once a fortnight. Hon. Secretary, Dr. W. H. St. John-Brooks, Johns Corner, Marazion.

### LIST OF CLUBS AND SOCIETIES WITH NAMES OF HON. SECRETARIES

### *British Medical Association, Branch and Division*

| | |
|---|---|
| *Bath, Bristol and Somerset* .. | Dr. K. C. Bailey, Tone Vale House, Norton Fitzwarren, nr. Taunton, Somerset. |
| Bath .. .. .. .. | Dr. E. Scott White, 2 Green Park, Bath, Somerset. |
| Bristol .. .. .. .. | Dr. R. C. Wofinden, 14 Upper Belgrave Road, Bristol 8.<br>Dr. W. H. Hayes, 70 Coombe Lane, Westbury-on-Trym, Bristol. |
| East Somerset .. .. | Dr. Mary A. E. Somers, 2 Ashcombe Road, Weston-super-Mare, Somerset. |
| West Somerset .. .. | Dr. J. Benn, 9 Haines Hill, Taunton. |
| *Gloucestershire* .. .. | Dr. E. W. Hyde, X-ray Department, Royal Infirmary, Gloucester.<br>Dr. D. C. Reavell, 50 London Road, Gloucester. |
| *South Western* .. .. | Mr. J. D. R. Murray, 12 Carlton Hill, Exmouth. |
| Barnstaple .. .. .. | Dr. S. G. Brook, 19 Bear Street, Barnstaple. |
| Cornwall .. .. .. | Dr. Eric Townsend, 22 Basset Road, Camborne, Cornwall. |
| Exeter .. .. .. .. | Dr. H. S. Gaussen, 6 Elm Grove Road, Exeter.<br>Mr. W. H. Rowe Jeremy, 38 Barnfield Road, Exeter. |
| Plymouth .. .. .. | Dr. G. Deery, 7 Hartley Park Gardens, Plymouth.<br>Mr. R. Howarth, St. Mary's, Plympton, Devon. |
| Torquay .. .. .. | Dr. Angus Everard, 6 St. Paul's Road, Newton Abbot. |
| *Wiltshire:* | |
| Trowbridge .. .. .. | Dr. P. A. H. Rivett, 5 Market Hill, Calne, Wilts. |

### MEDICAL SOCIETIES (GENERAL)

| | |
|---|---|
| The Clinical Society of Bath | Mr. A. D. Bateman, 3 The Circus, Bath. |
| Cornwall Clinical Society .. | Dr. L. W. Hale, Penvu, Camborne, Cornwall.<br>Dr. F. D. M. Hocking, St. Andrews, Perranporth, Cornwall. |
| West Penwith Medical Society | Dr. W. H. St. John-Brooks, Johns Corner, Marazion. |
| Torquay and District Medical Society. | Dr. R. A. Lattey, Ambrook, Rousdown Road, Torquay. |
| Devon and Exeter Medico-Chirurgical Society. | Dr. C. M. Seward, 20 West Southernhay, Exeter. |
| The Plymouth Medical Society | Mr. W. B. Waterfall, 2 Crescent Villas, Athenaeum Street, Plymouth. |
| Weston-super-Mare Medical Society. | Dr. J. D. Powell, 2 St. Paul's Road, Weston-super-Mare, Som. |
| Bristol Medico-Chirurgical Society. | Mr. A. L. Eyre-Brook, Litfield House, Clifton, Bristol 8. |
| Cossham Medical Society .. | Dr. I. Macdonald, 43 Regent Street, Kingswood, Bristol. |
| Bishopston General Practitioners Group. | Dr. I. L. Maxwell, 237 Gloucester Road, Bristol. |
| Bristol South General Practitioners Group. | Dr. D. M. Cameron, Lindthorpe, 1 Dean Lane, Southville, Bristol 3. |
| Westbury General Practitioners Group. | Dr. J. Mason, 7 Westbury Road, Westbury-on-Trym, Bristol 9 |

